# Pathological complete response after neoadjuvant chemotherapy with trastuzumab-containing regimen in gastric cancer: a case report

**DOI:** 10.1186/1756-8722-3-31

**Published:** 2010-09-09

**Authors:** Jun Wang, George W Saukel, Carlos A Garberoglio, Wichit Srikureja, Chung-Tsen Hsueh

**Affiliations:** 1 Department of Pathology and Laboratory Medicine, Loma Linda University Medical Center, Loma Linda, CA 92354, USA; 2 Department of General and Trauma Surgery, Loma Linda University Medical Center, Loma Linda, CA 92354, USA; 3 Division of Gastroenterology, Loma Linda University Medical Center, Loma Linda, CA 92354, USA; 4 Division of Medical Oncology and Hematology, Loma Linda University Medical Center, Loma Linda, CA 92354, USA

## Abstract

We report a 49-year-old Chinese male with locally advanced gastric adenocarcinoma achieving pathological complete response after neoadjuvant chemotherapy with trastuzumab-containing regimen. He underwent esophagogastroduodenoscopy in September 2009, which revealed a 2-cm gastric ulcer on the lesser curvature proximal to angularis. Biopsy of gastric ulcer showed moderately differentiated adenocarcinoma with overexpression of human epidermal growth factor receptor 2 (HER2) by immunohistochemistry and fluorescence in situ hybridization. Further workups with endoscopic ultrasound, computed tomography and positron emission tomography staged his cancer as T3N1M0. He received 3 cycles of neoadjuvant chemotherapy consisting of trastuzumab, oxaliplatin, docetaxel and capecitabine without severe toxicities except grade 2 diarrhea near the completion of cycle 3 requiring discontinuation of capecitabine. Afterwards, he received total gastrectomy with extended D2 lymph node dissections showing pathological complete response. He went on to receive 3 more cycles of chemotherapy postoperatively. The role of trastuzumab as a part of perioperative therapy in gastric cancer overexpressing HER2 is worth further investigation.

## Introduction

Gastric cancer is the fourth most common cancer worldwide, with overall 5-year survival rate of approximate 20%, representing a significant challenge for the treating physicians [1]. Perioperative chemotherapy has been shown to cause tumor downstaging and improve survival in patients with resectable gastric cancer [2]. Response to neoadjuvant treatment is the most important predictor of survival after curative resection of gastric cancer [3-5].

In this case report, we describe a case of pathological complete response after neoadjuvant chemotherapy with trastuzumab-containing regimen in gastric cancer. We discuss histopathological findings and review the pertinent literatures.

## Case report

**Figure 1:**
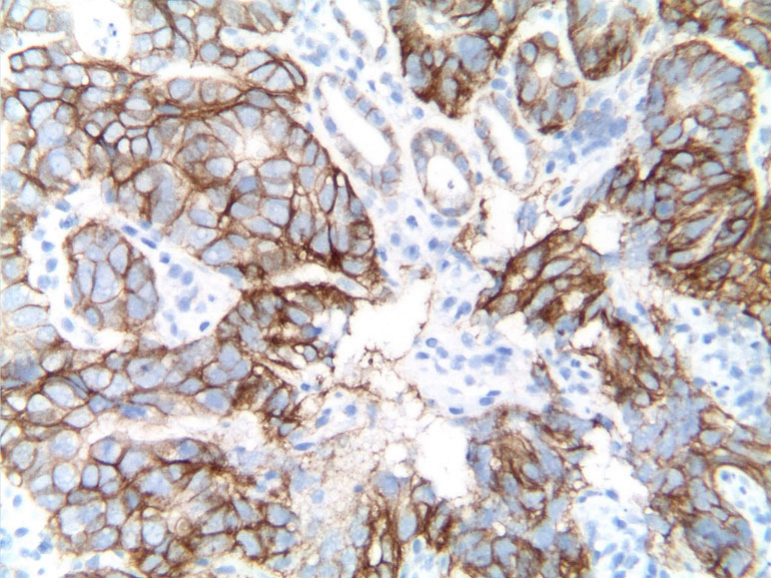
**Immunohistochemical study of HER2 protein in biopsied specimen before chemotherapy**. The moderately differentiated adenocarcinoma cells infiltrated the gastric submucosa and overexpressed HER2 (3+ by HercepTest) on the cell membrane (immunoperoxidase stain, 200×).

A 49-year-old Chinese male with gastroesophageal reflux disease and H. Pylori infection underwent esophagogastroduodenoscopy (EGD) in September 2009, which revealed a 2-cm gastric ulcer on the lesser curvature proximal to angularis. Biopsy of gastric ulcer showed moderately differentiated adenocarcinoma. Tumor analysis for human epidermal growth factor receptor 2 (HER2) was performed by HercepTest (Genzyme, Los Angeles, CA) indicating 3+ immunohistochemistry (IHC) staining (Fig. 1). HER2 gene amplification was confirmed by fluorescence in situ hybridization (FISH) demonstrating HER2/CEP17 (chromosome enumeration probe 17) ratio of 4. Endoscopic ultrasound study indicated presence of perigastric lymphadenopathy and tumor invading through the muscularis propria. Other staging workups, including computed tomography (CT) scan of chest, abdomen and pelvis and positron emission tomography-CT (PET-CT) scan, did not reveal any distant metastasis. The clinical staging was T3N1M0, and patient was recommended to receive neoadjuvant chemotherapy before definitive surgery. The information of ToGA trial was presented to patient [6], and patient agreed to receive trastuzumab-containing regimen: trastuzumab 6 mg/kg iv on day 1, oxaliplatin 130 mg/m^2 ^iv on day 1, docetaxel 30 mg/m^2 ^iv on day 1 and day 8, and capecitabine 625 mg/m^2 ^po bid on day 1 to day 21, every 3 weeks. He received 3 cycles chemotherapy without severe toxicities except grade 2 diarrhea near the completion of cycle 3 requiring discontinuation of capecitabine. The post-treatment imaging studies including CT scan of chest, abdomen and pelvis and PET-CT scan showed persistent mild FDG [fluorodeoxyglucose (18F)] activity involving the stomach without identifiable mass or distant metastasis.

**Figure 2:**
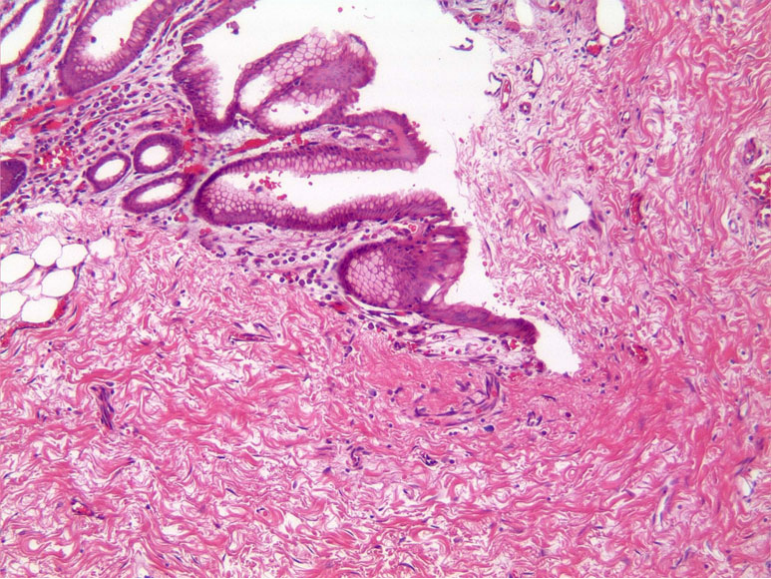
**Microscopic finding of the resected specimen after chemotherapy**. No residual adenocarcinoma was found in the original ulcerated adenocarcinoma site on lesser curvature. Instead, it was completely replaced by dense fibrous tissue with partial re-epithelialization of the overlying mucosal surface and fibrosis extending into muscularis propria (hematoxylin and eosin stain, 100×).

In January 2010, he received total gastrectomy with extended D2 lymph node dissections, Roux-en-Y esophagojejunostomy and cholecystectomy. Prior to surgical resection, the intraoperative EGD showed a healed scar in the original ulcerative tumor site, and laparoscopy revealed no evidence of peritoneal carcinomatosis or metastatic implants. Pathological examination of the surgical specimen indicated no residual adenocarcinoma but scar on lesser curvature with fibrosis extending into muscularis propria (Fig. 2). There were no tumor identified in 44 perigastric lymph nodes and 2 lymph nodes from porta hepatis. He recovered uneventfully after surgery, and received 3 more cycles of chemotherapy with the same regimen with dose reduction on docetaxel and capecitabine due to gastrointestinal toxicities. He has remained free of disease after completion of chemotherapy.

## Discussion

HER2 exhibits tyrosine kinase activity and functions as a growth factor receptor [7]. HER2 overexpression due to gene amplification in gastric cancer has led to aggressive clinical course and poor prognosis [8]. Trastuzumab, a monoclonal antibody against HER2, causes cell cycle arrest at G1 and exhibits antitumor activity in HER2 overexpressed gastric cancer cells [9,10]. Additionally, trastuzumab can enhance cytotoxic effects of chemotherapy in gastric cancer xenograft overexpressing HER2, when combined with capecitabine, cisplatin, or taxane [11]. Phase II studies incorporating trastuzumab with cisplatin-based regimen in patients with advanced gastric cancer overexpressing HER2 have demonstrated promising activities [12,13].

The ToGA study presented at 2009 annual meeting of American Society of Clinical Oncology has screened about 3,800 patients with advanced gastric cancer from 24 countries [14]. HER2 overexpression was detected in 22%, and the concordance rate between IHC and FISH was high at all levels of HER2 positivity [15]. There was a specific pattern of disease which correlated with HER2 overexpression. Higher rates occurred in intestinal and proximal or gastroesophageal junction cancers than in diffuse or distal gastric cancers.

Five hundred and eighty four patients tested positive for HER2 overexpression (IHC 3+ and/or FISH positive) were enrolled into ToGA study, a phase III trial comparing fluoropyrimidine (5-fluorouracil [5-FU] or capecitabine) and cisplatin chemotherapy with or without trastuzumab. Patients who received trastuzumab plus chemotherapy achieved longer overall survival (13.8 months vs. 11.1 months, P = 0.0046), longer progression-free survival (6.7 months vs. 5.5 months, P = 0.0002), and higher response rates (47% vs. 35%, P = 0.0017) than those who received chemotherapy alone. Complete response was noted in 5.4% of patients receiving trastuzumab plus chemotherapy vs. 2.4% in chemotherapy alone. There were no significant differences in the toxicities between these two groups. This study has established a new paradigm using trastuzumab in combination with chemotherapy in patients with advanced gastric cancer overexpressing HER2.

MAGIC trial for investigation of perioperative chemotherapy was conducted in patients with resectable adenocarcinoma of stomach, gastroesophageal junction or distal esophagus [2]. Five hundred and three patients were randomly assigned to either perioperative chemotherapy with epirubicin, cisplatin and infusional 5-FU (ECF) and surgery or surgery alone. Despite of only 43% of patients completing the planned 6 cycles of chemotherapy (3 cycles before surgery and 3 cycles afterwards), there was statistically significant improvement in overall survival in patients receiving chemotherapy and surgery (5-year survival: 36% for chemotherapy plus surgery vs. 23% for surgery). At the time of surgery, the patients receiving preoperative chemotherapy had significantly smaller tumor size and lower stage. However, there was no pathological complete response in patients receiving preoperative ECF in this study.

The infusional 5-FU in the ECF regimen is given continuously through a venous access device, and is associated with inconvenience and higher incidence of thrombosis and infection. Furthermore, cisplatin can cause nephrotoxicity, ototoxicity, and severe emesis. REAL-2, a randomized study in patients with advanced gastroesophageal cancer using two-by-two design, has shown 5-FU can be replaced by capecitabine, and cisplatin by oxaliplatin in the regimen of ECF without affecting the efficacy [16].

Docetaxel has demonstrated encouraging activity in the treatment of advanced gastric cancer [17]. Phase II/III trial V325 has shown adding docetaxel to cisplatin and 5-FU (DCF) significantly improved time to tumor progression, survival, and response rate in advanced gastric cancer patients receiving first-line treatment [18]. Based on results of this study, Food and Drug Administration of U.S.A. approved DCF for the treatment of advanced gastric cancer in 2006. Various modifications of DCF with the intent to improve tolerability have been developed. Replacing cisplatin by oxaliplatin and 5-FU by capecitabine, the combination of docetaxel, oxaliplatin and capecitabine have demonstrated encouraging activity and good tolerability in early-phase studies [19,20].

The administration of trastuzumab can result in sub-clinical and clinical cardiac failure, and the incidence is much higher in patients receiving trastuzumab concurrently with anthracycline-containing chemotherapy regimens [21]. Instead of using ECF, we decided to use docetaxel, oxaliplatin and capecitabine in combination with trastuzumab as perioperative chemotherapy for our patient mainly due to the concern of cardiac toxicity. We have monitored our patient's cardiac function with periodic echocardiogram evaluation, and find no evidence of cardiac failure.

Our case illustrates the first reported case of pathological complete response after neoadjuvant chemotherapy with trastuzumab-containing regimen in a patient with locally advanced gastric cancer overexpressing HER2. The use of docetaxel, oxaliplatin and capecitabine in combination with trastuzumab in this setting remains experimental, and ideally should be considered only in the context of a clinical trial. Therefore, the role of trastuzumab as a part of perioperative therapy is worth further investigation.

## Consent

Written informed consent was obtained from each patient for publication of this case report and accompanying images. A copy of the written consent is available for review by the Editor-in-Chief of this journal.

## Competing interests

The authors declare that they have no competing interests.

## Authors' contributions

CTH designed the paper. JW and CTH wrote the paper. WS performed endoscopic examination and biopsy. CG performed surgery. JW and GWS provided pathological evaluation. All authors read and approved the final manuscript.
